# The Role of Proinflammatory Pathways in the Pathogenesis of Colitis-Associated Colorectal Cancer

**DOI:** 10.1155/2017/5126048

**Published:** 2017-08-09

**Authors:** Chengxin Luo, Hu Zhang

**Affiliations:** Department of Gastroenterology, West China Hospital, Sichuan University, Chengdu, China

## Abstract

Patients with inflammatory bowel disease (IBD) are at an increased risk of developing colorectal cancer (CRC). The risk factors of CRC in IBD patients include long disease duration, extensive colitis, severe histological inflammation, and coexistence with primary sclerosing cholangitis (PSC). Several molecular pathways that contribute to sporadic CRC are also involved in the pathogenesis of colitis-associated CRC. It is well established that long-standing chronic inflammation is a key predisposing factor of CRC in IBD. Proinflammatory pathways, including nuclear factor kappa B (NF-*κ*B), IL-6/STAT3, cyclooxygenase-2 (COX-2)/PGE_2_, and IL-23/Th17, promote tumorigenesis by inducing the production of inflammatory mediators, upregulating the expression of antiapoptotic genes, and stimulating cell proliferation as well as angiogenesis. Better understanding of the underlying mechanisms may provide some promising targets for prevention and therapy. This review aims to elucidate the role of these signaling pathways in the pathogenesis of colitis-associated CRC using evidence-based approaches.

## 1. Introduction

Inflammatory bowel disease (IBD), including ulcerative colitis (UC) and Crohn's disease (CD), is a chronic incurable disease that can affect the entire gastrointestinal tract. One of the most serious complications of IBD is colorectal cancer (CRC). Patients with IBD are at a higher risk of developing CRC compared to the general population [1]. The incidence rate of CRC in the general population ranges from 0.1/1000 person-years (py) to 0.4/1000 py. A meta-analysis including 116 studies (54,478 patients) estimated that the overall incidence rate of CRC in patients with UC was 3/1000 py. This risk increased with disease duration, which was 2% at 10 years, 8% at 20 years, and 18% at 30 years [2]. More recently, meta-analysis published in 2013 suggested that the incidence rate of CRC in UC patients decreased to 1.58/1000 py due to more widespread endoscopic surveillance, the use of chemopreventive agents such as 5-aminosalicylates (5-ASA), and higher rates of colectomy [3]. The role of CD in CRC risk remains controversial [4, 5]. Although some studies failed to confirm an association, meta-analysis conducted by Canavan et al. revealed a significant increased risk of CRC in CD, with a relative risk (RR) of 2.5 (95% CI 1.3–4.7) [4]. The cumulative risk of CRC in patients diagnosed with CD in this study was estimated as 2.9% at 10 years, 5.6% at 20 years, and 8.3% at 30 years. Subgroup analysis demonstrated no significant difference in risk between patients with ileal disease and the general population. In contrast, the risk increased by at least fourfold in patients with colonic disease [4]. These data demonstrate that repeated inflammation is a risk factor for CRC in patients with IBD.

In addition to long disease duration, extensive colitis is a risk factor for the development of CRC in IBD. The risk of CRC dramatically increased in patients with extensive disease [6, 7]. According to a meta-analysis, the cumulative incidence of CRC in patients with extensive colitis was 44.4/1000 patients [3]. A significant correlation between the histological inflammation score and the risk of developing CRC in IBD patients has been demonstrated, which suggests that severity of inflammation is an independent risk factor [8]. Primary sclerosing cholangitis (PSC), a progressive cholestatic hepatobiliary inflammatory disease, is also an independent risk factor as coexistence of PSC increased the risk of CRC by approximately fourfold compared to patients with UC alone [9, 10]. 5-ASA compounds, the anti-inflammatory drugs that are most widely used in the management of IBD, have been reported to decrease the risk of CRC [5]. To some extent, 5-ASA compounds function as chemopreventive agents, suggesting that inflammation plays a key role in the pathogenesis of CRC in IBD patients.

The pathogenesis of CRC in IBD patients involves genetic and epigenetic changes. Sporadic CRC and colitis-associated CRC share some common genetic changes, including the inactivation of tumor suppressor genes and mutation in oncogenes as well as genetic instability [11]. However, differences in timing and the sequence of these molecular alterations are present. For example, the mutation in *adenomatous polyposis coli* (APC) was found in colitis-associated CRC but is less frequent than that observed in sporadic CRC and occurs later [12]. In addition, differences in morphology and biological behavior between colitis-associated CRC and sporadic CRC have been demonstrated, such as increased prevalence of multifocal invasive lesions, higher rates of mucinous or signet-ring cell carcinomas, and poor survival in colitis-associated CRC [13]. The typical “normal mucosa-adenoma-dysplasia-carcinoma” sequence in the sporadic CRC development was not confirmed in colitis-associated CRC, which arises in inflamed mucosa and develops in an “inflammation-dysplasia-carcinoma” sequence [11]. IBD is characterized by chronic inflammation in the mucosa. It is well recognized that the long-standing chronic inflammation in the mucosa contributes to the occurrence of carcinoma. The degree of inflammation and duration of disease are closely related with the risk of CRC. On the other hand, anti-inflammatory drugs are protective against the development of CRC. It is suggested that signals activated in chronic inflammation may contribute to tumorigenesis through increasing oxidative stress, promoting epithelial cell proliferation, and supporting angiogenesis [14, 15]. This review aims to elucidate the role of chronic inflammation in colitis-associated CRC with a review regarding the contribution of inflammatory signaling pathways, including nuclear factor kappa B (NF-*κ*B), IL-6/STAT3, cyclooxygenase-2 (COX-2)/PGE_2_, and IL-23/Th17.

## 2. NF-*κ*B Pathway

**Figure 1:**
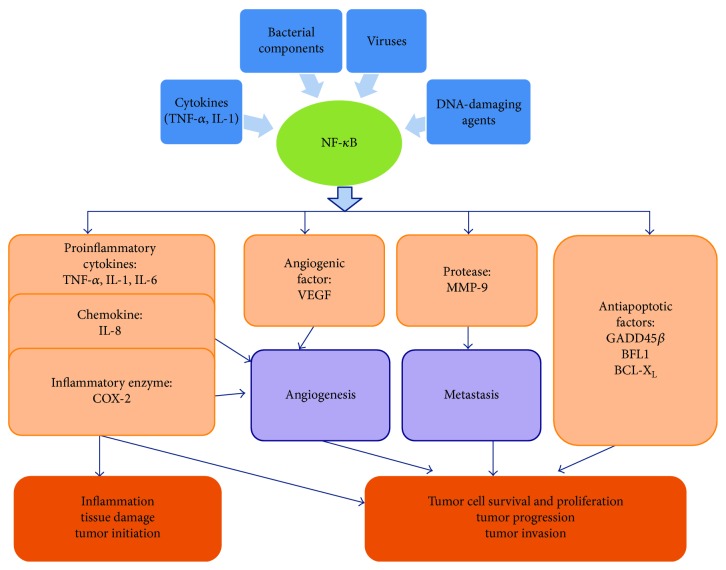
NF-*κ*B pathway functions as the molecular link between inflammation and tumorigenesis. NF-*κ*B pathway can be activated by proinflammatory cytokines (TNF-*α* and IL-1), bacterial components (such as LPS), viruses, and DNA-damaging agents. Activation of NF-*κ*B pathway induces expression of proinflammatory cytokines (such as TNF-*α*, IL-1, and IL-6), chemokines (IL-8), and enzymes (COX-2) that contribute to inflammation-related tissue damages and are associated with tumor initiation. Antiapoptotic factors GADD45*β*, BFL1, and BCL-X_L_ ensure tumor cell survival and proliferation. In addition, VEGF, COX-2, and IL-8 promote angiogenesis and play an important role in tumor progression. MMP-9 contributes to tumor progression through facilitating metastasis.

NF-*κ*B is a key regulator of inflammation and can be activated by a broad panel of stimuli, including bacterial components such as lipopolysaccharide (LPS), proinflammatory cytokines such as TNF-*α* and IL-1, viruses, and DNA-damaging agents [16]. Once activated, NF-*κ*B-bound I*κ*Bs (inhibitor of NF-*κ*B) are phosphorylated by the I*κ*B kinase (IKK) complex. Degradation of I*κ*Bs allows for NF-*κ*B to translocate to the nucleus and mediate the transcription of various target genes [17]. Some proinflammatory cytokines (such as TNF-*α*, IL-1, and IL-6) that are encoded by target genes of the NF-*κ*B pathway contribute to inflammation-related tissue damage and are associated with tumor development and progression. For example, TNF-*α* has been demonstrated as a potent mutagen that contributes to tumor initiation via the induction of reactive oxygen species (ROS) production and promoting DNA damage [18]. Increased expression and activation of NF-*κ*B were observed in IBD patients, especially in mucosal macrophages and epithelial cells, accompanied by enhanced production of proinflammatory cytokines such as TNF-*α*, IL-1, and IL-6 [19]. Target genes of the NF-*κ*B pathway that encode antiapoptotic regulators including grow arrest and DNA-damage-inducible 45*β* (GADD45*β*), B-cell lymphoma 2- (BCL-2-) related protein (BFL1), and B-cell lymphoma X_L_ (BCL-X_L_) have also been identified [16, 17]. It is well accepted that the activation of these antiapoptotic genes ensures the survival and proliferation of tumorigenic cells [17, 20]. In addition, activation of NF-*κ*B can stimulate tumor progression and invasion through directly or indirectly enhancing the expression of vascular endothelial growth factor (VEGF), COX-2, and IL-8 to promote angiogenesis [21]. Moreover, the production of matrix metalloproteinase- (MMP-) 9 and some serine proteases that are regulated by NF-*κ*B pathway was shown to facilitate tumor metastasis [16]. In conclusion, the NF-*κ*B pathway functions as a molecular link between inflammation and tumorigenesis due to its ability to stimulate the expression of proinflammatory cytokines, antiapoptotic genes, angiogenesis factors, and proteases, which promote tumor initiation and ensure the survival and proliferation as well as invasion of malignant cells (Figure 1).

An association between the NF-*κ*B pathway and the pathogenesis of colitis-associated CRC was confirmed in animal experiments. An animal model of colitis-associated CRC was established with injection of a procarcinogen azoxymethane (AOM), followed by repeated oral administration of dextran sulfate sodium (DSS), which causes chronic inflammation mimicking IBD. All mice treated with this protocol developed tumors in the middle to distal colon, where the most severe inflammation occurs in DSS-induced colitis [22]. Enterocyte-specific deletion of IKK*β* significantly decreased the incidence of colitis-associated tumors, although a significant increase in levels of histological inflammation and proinflammatory cytokines was observed. Administration of AOM and DSS led to the activation of IKK and induction of the antiapoptotic protein BCL-X_L_, which is absent in the IKK*β*-knockout mice. Deletion of IKK*β* in enterocytes also increased apoptosis through upregulating the expression of proapoptotic proteins Bak and Bax. These results suggested that the NF-*κ*B pathway in epithelial cells promotes tumor development by suppressing apoptosis rather than mediating the transcription of proinflammatory genes [16, 22]. However, myeloid-specific deletion of IKK*β* significantly decreased the incidence and size of tumors in colitis-associated cancer model without an effect on apoptosis. Deletion of IKK*β* in myeloid cells reduced the expression of proinflammatory mediators and the proliferation of epithelial cells [22]. Collectively, the NF-*κ*B pathway does promote tumor growth in animal models but the pathway to suppress apoptosis or induce proinflammatory cytokines may be cell type dependent.

Activation of NF-*κ*B induces the production of TNF-*α*, which can further increase NF-*κ*B activation after binding to TNF receptor (TNF-R). Crucial contributions of TNF-*α*/TNF-R in tumor initiation and progression have been suggested [23]. In addition to promoting DNA damage by inducing ROS generation, several lines of evidence have implied that TNF-*α* promotes angiogenesis via stimulating the expression of proangiogenic chemokines, which can induce endothelial cell proliferation by increasing the recruitment of inflammatory cells that secrete angiogenic factors [24]. Enhanced expression of TNF-*α* was demonstrated in colitis-associated CRC mouse models that were established by combined treatment of AOM and DSS. Knockout of TNF-Rp55 (TNF receptor p55) or treatment with TNF-*α* antagonist etanercept reduced mucosal inflammatory cell infiltration, tumor incidence, and tumor size [23]. Infliximab, a novel anti-TNF-*α* compound that is used in the management of patients with refractory IBD, was suggested to be effective in cancer prevention with early intervention in animal models of colitis-associated CRC [25]. Above all, TNF-*α* is a key risk factor within the NF-*κ*B pathway to the development of colitis-associated CRC.

## 3. IL-6/STAT3 Pathway

The proinflammatory cytokine IL-6 plays a crucial pathogenic role in IBD. The levels of IL-6 in serum and intestinal mucosa of patients with IBD are distinctly elevated and positively correlated with the severity of inflammation [26]. The classic IL-6 pathway is initiated by binding to its membrane-bound receptor (IL-6R) to form an IL-6/IL-6R complex, which induces the recruitment and homodimerization of two gp130 subunits to activate intracellular signal pathways, including the JAK/STAT, Ras/ERK (extracellular signal-regulated kinase), and PI3K (phosphoinositide 3-kinase)/Akt pathway. The gp130 protein is ubiquitously expressed. In cells that do not express IL-6R and only express gp130, IL-6 can bind to a soluble form of IL-6R (sIL-6R) to initiate intracellular signaling, termed transsignaling [27]. Several studies have demonstrated that proinflammatory activities of IL-6 are mediated via transsignaling [28–30]. Increased levels of circulating sIL-6R and IL-6/sIL-6R complexes in IBD patients have been observed. In IBD, activation of STAT3 that is mediated by the interaction of IL-6/sIL-6R complexes and gp130 enhances the expression of antiapoptotic factors such as BCL-2 and BCL-X_L_, which causes apoptotic resistance in CD4^+^T cells and contributes to the perpetuation of chronic intestinal inflammation [26]. A crucial role of IL-6 in the pathogenesis of CRC has been suggested as serum IL-6 levels were significantly increased in patients with CRC and correlated with tumor size and disease status [31]. The IL-6/STAT3 pathway can stimulate the survival and proliferation of premalignant intestinal epithelial cells (IEC) [32]. Recent studies have demonstrated that the IL-6/STAT3 pathway is a crucial tumor promoter in colitis-associated cancer.

In mouse models of colitis-associated cancer that combined the treatment of AOM and DSS, IL-6 was mainly expressed by infiltrating macrophages, dendritic cells, and T cells during tumorigenesis [32, 33]. Deletion of IL-6 reduced tumor numbers, tumor size, and tumor multiplicity. IL-6^−/−^ mice exhibited a more severe DSS-induced colitis, elevated apoptosis, and decreased IEC proliferation, similar to mice with IEC-specific deletion of STAT3 [32, 34]. STAT3 was highly activated in DSS-induced colitis, IBD, and various tumors [35, 36]. Some target genes of STAT3 signaling are important for cell proliferation, such as cyclin D and PCNA (proliferating cell nuclear antigen), which were downregulated in IL-6^−/−^ mice [32, 36]. Expression of BCL-X_L_ was also downregulated when IL-6 or STAT3 was ablated [32]. Collectively, it is suggested that IL-6 is a critical tumor promoter in colitis-associated cancer and STAT3 is essential for the transduction of tumor-promoting signals from IL-6.

SOCS3 (suppressor of cytokine signaling 3), a STAT3 target gene, is a negative regulator of the IL-6/STAT3 pathway [37]. *In vitro*, overexpression of SOCS3 reduced IL-6-dependent STAT3 activation. SOCS3-positive cells were significantly increased in colonic epithelium of patients with active IBD, together with increased expression of IL-6 and phosphorylated STAT3 (p-STAT3). In contrast, decreased SOCS3 expression and methylation of SOCS3 were observed in patients with colitis-associated CRC [38]. In AOM/DSS models of colitis-associated cancer, IEC-specific deletion of SOCS3 enhanced crypt proliferation and promoted tumor growth [39]. These results implied an important role of SOCS3 in inhibiting tumorigenesis via downregulating IL-6/STAT3 signaling.

In addition, a link between TGF-*β* signaling and IL-6/STAT3 signaling in the tumorigenesis of colitis-associated CRC has been demonstrated. Several lines of evidence support a protective role of TGF-*β* in the development of CRC [5, 40]. Mutations in the TGF-*β* receptor II (TGF-*β*RII) were detected in patients with CRC [41]. In AOM/DSS-treated mice, decreased expression of TGF-*β*R was observed in dysplastic epithelial cells although large amounts of TGF-*β* were expressed by tumor infiltrating T cells. In the same animal model, overexpression of TGF-*β* reduced IL-6 production, delayed tumor development, and inhibited tumor growth. In contrast, dominant-negative TGF-*β*RII transgenic mice exhibited increased tumor burden, which could be inhibited by neutralization of IL-6R [33]. It is suggested that suppression of TGF-*β* signaling promotes tumor growth in an IL-6/STAT3-dependent way.

## 4. COX-2/PGE_2_ Pathway

Evidences from population-based studies and animal experiments support a protective role of nonsteroidal anti-inflammatory drugs (NSAIDs) against CRC [42]. Long-term use of NSAIDs reduced the risks of developing CRC by 40–50% [43]. NSAIDs inhibit the activity of COX, the enzyme that catalyzes the formation of prostaglandins (PGs). Two isoforms of COX enzyme have been cloned; COX-1 is constitutively expressed in various cells while COX-2 is not normally expressed but can be induced by growth factors and proinflammatory cytokines [44]. The anticancer effects of NSAIDs are due to their ability to inhibit the inducible COX-2.

COX-2 plays an important role in colonic inflammation and tumorigenesis. Elevated COX-2 expression was observed in approximately 85% of CRCs and correlated with poorer survival [44]. In IBD, COX-2 overexpression was detected in patients with active inflammation and in colitis-associated neoplastic tissues [45]. In animal models, including Apc^Min^ mice and AOM-treated mice, deletion of COX-2 or treatment with selective COX-2 inhibitors reduced tumor numbers, size, and multiplicity [44, 46, 47]. COX-2 may promote tumor development through its ability to induce the expression of antiapoptotic proteins such as BCL-2 and result in resistance to apoptosis. In addition, overexpression of COX-2 is associated with elevated levels of MMPs and increased migration of malignant cells [44].

As downstream of COX-2, PGE_2_ mediates the effects of COX-2 in IBD and CRC. PGE_2_ acts via a specific cell surface receptor EP, which is comprised of four subtypes, EP1, EP2, EP3, and EP4 [48]. The proinflammatory effects of PGE_2_ are primarily mediated through EP1 and EP3. However, recent studies have demonstrated that PGE_2_ can interact with EP2/EP4 receptors on dendritic cells to induce the expression of IL-23 and exacerbate experimental colitis [49]. EP4, a PGE_2_ receptor subtype that is constitutively expressed in the colonic epithelium, plays an important role in epithelium survival and regeneration through the activation of antiapoptotic as well as proliferative signaling pathways. Exacerbated DSS-induced colitis was observed after deletion of EP4 [50]. *In vitro*, administration of PGE_2_ induces BCL-2 expression, decreases apoptosis, and promotes proliferation in human CRC cells [51]. A selective agonist of EP4 promotes colon cancer cell growth at the same level as PGE_2_. Knockout of EP4 or administration with a selective EP4 antagonist decreased AOM-induced preneoplastic lesions and intestinal polyp numbers [52]. It is suggested that EP4 can mediate cancer-promoting effects of PGE_2_ in CRC. Moreover, a key role of EP1 in colon carcinogenesis has also been demonstrated with AOM-treated mice, in which genetic or pharmacological deletion of EP1 significantly inhibited tumor development [53]. Furthermore, PGE2 can promote tumor progression by inducing the expression of C-X-C motif ligand 1 (CXCL1), a proangiogenic chemokine that can induce microvascular endothelial cell migration and tube formation to promote tumor growth as well as invasion [54].

**Figure 2:**
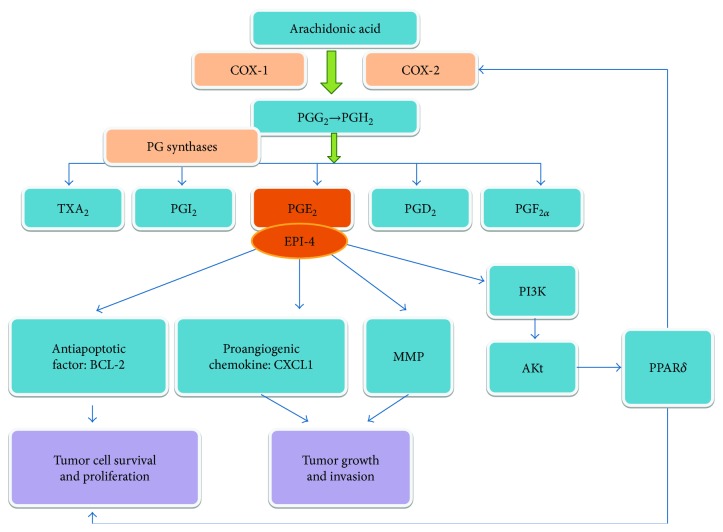
The role of COX-2/PGE_2_ pathway in colitis-associated CRC. The COX enzymes catalyze the biosynthesis of PGG_2_ and PGH_2_ from arachidonic acid. PGH_2_ is subsequently metabolized to TXA_2_, PGI_2_, PGE_2_, PGD_2_, and PGF_2*α*_ by PG synthases. COX-2-derived PGE_2_ acts via specific receptor EP1-4 and plays an important role in tumor development and progression through inducing expression of antiapoptotic proteins (such as BCL-2), proangiogenic chemokines (such as CXCL1), and MMP. In addition, PGE_2_ mediates the activation of PPAR*δ* through PI3K/Akt signaling. PPAR*δ* contributes to tumorigenesis by ensuring tumor cell survival and proliferation. Activation of PPAR*δ* can further enhance the production of COX-2-derived PGE_2_.

Additionally, transactivation of nuclear hormone receptor peroxisome proliferator-activated receptors (PPARs) is involved in the cancer-promoting effects of PGE2. In Apc^min^ mice, PGE_2_ treatment promoted cell survival and distinctly increased tumor burden, which was mediated by the transactivation of PPAR*δ* through PI3K/Akt signaling [55]. PPAR*δ* is one of the downstream targets of the COX-2/PGE_2_ pathway. However, activation of PPAR*δ* can induce COX-2 expression in colonic cancer cells. COX-2-derived PGE_2_ stimulates macrophages to produce proinflammatory cytokines that contribute to colitis-associated cancer. Deletion of PPAR*δ* attenuated colonic inflammation and colitis-associated tumor development in animal experiments [56]. It is suggested that PGE_2_ mediates the crosstalk between colonic tumorigenesis and chronic inflammation via a self-amplifying loop between PPAR*δ* and the COX-2/PGE_2_ pathway (Figure 2).

## 5. IL-23/Th17 Pathway

Recent studies have shown that a novel T-cell subset, T-helper IL-17-producing (Th17) cell, is involved in the pathogenesis of IBD [57]. The IL-17 cytokine family is comprised of six members: IL-17A, IL-17B, IL-17C, IL-17D, IL-17E (also known as IL-25), and IL-17F. IL-17A and IL-17F drive intestinal inflammation by inducing cytokine and chemokine production from endothelial cells and macrophages as well as increasing neutrophil recruitment [58]. Increased IL-17 expression was detected in the serum and colonic mucosa in patients with active IBD [59]. Additionally, Th17 cells can also synthesize other cytokines that have been shown to play an important role in intestinal inflammation, such as IL-21 and IL-22. IL-23, a heterodimeric cytokine composed of a p19 and p40 subunit, is a positive regulator of Th17 cells. TGF-*β* and IL-6 drive early Th17 cell differentiation by promoting the expression of the crucial transcription factor retinoic acid receptor-related orphan receptor (ROR) *γ*t and ROR*α*. TGF-*β* and IL-6 can also induce IL-23 receptor (IL23R) expression on Th17 cells to mediate the effects of IL-23 [60]. IL-23 is required in the stabilization and expansion of the Th17 response. Blockade of IL-23 signaling with monoclonal anti-IL-23p19 antibody induced apoptosis of Th17 cells and attenuated experimental colitis induced by transferring IL-17-producing CD4^+^ T cells to SCID mice [61]. In addition, genetic studies have demonstrated that variants of the *IL23R* gene are linked to IBD susceptibility [62]. So far, it is well known that the IL-23/Th17 pathway plays a key role in the pathogenesis of IBD [57, 58, 60].

IL-23/Th17 signaling not only contributes to inflammation in IBD but also enhances tumor growth and progression in CRC. In CPC-APC mice (the Apc^F/wt^ mice that harbor a Cdx2-Cre transgene and primarily develop tumors in the distal colon), upregulation of IL-23 and IL-17A was observed in colonic tumors. Ablation of IL-23 or IL-23R attenuated the expression of IL-17A and reduced cell proliferation and tumor load [63]. Furthermore, the number of IL-17-producing cells is positively correlated with intratumoral microvessel density. It is suggested that IL-17 facilitates angiogenesis and promotes CRC development by inducing the production of VEGF [64]. Similarly, the level of serum IL-23 was elevated in patients with CRC and correlated with the expression of VEGF [65]. The critical role of IL-17 in the pathogenesis of colitis-associated cancer was also confirmed in animal models. Blocking IL-17A attenuated colitis and reduced tumor burden in APC^min/+^ mice and AOM/DSS-treated mice [66, 67].

Th17 cells can also produce large amounts of IL-21, which in turn amplifies Th17 cell responses by activating STAT3 and upregulating IL-23R [67]. The role of IL-21 in colitis-associated CRC has been investigated. IL-21 was found overexpressed in patients with UC and colitis-associated CRC. Deletion of IL-21 attenuated intestinal inflammation by reducing the infiltration of T cells, the activation of STAT3, and the production of IL-6 as well as IL-17A. Furthermore, deletion of IL-21 could further reduce tumor numbers and tumor size in AOM/DSS-treated mice [68]. IL-22, another cytokine secreted by Th17 cells, was also upregulated in infiltrated leukocytes in tumor masses of patients with colitis-associated CRC. These IL-22-producing cells can promote tumor growth and metastasis by activating STAT3 and inducing the expression of antiapoptotic factors such as BCL-X_L_ [69]. Deficiency in IL-22-binding protein (IL-22BP), a soluble receptor that can neutralize IL-22 to inhibit IL-22 signaling, enhanced tumorigenesis in APC^min/+^ mice and AOM/DSS-treated mice [70]. Collectively, Th17 cell cytokines IL-21 and IL-22 have tumor-promoting effects in colitis-associated CRC. In contrast, IL-17F played a protective role in colonic tumorigenesis as IL-17F-deficient mice exhibited an increased level of VEGF and developed more tumors after treatment with both AOM and DSS [71].

## 6. Conclusion

Perpetuated intestinal inflammation in IBD dramatically increases the risk of CRC. Clinical studies and animal experiments have demonstrated a crucial role of proinflammatory pathways, especially the NF-*κ*B, IL-6/STAT3, COX-2/PGE2, and IL-23/Th17 signaling pathways, in the pathogenesis of colitis-associated CRC. These signaling pathways regulate the expression of various inflammatory mediators and orchestrate a tumor-supporting microenvironment. The tumor-promoting effects of these signals involve the upregulation of antiapoptotic proteins and the enhanced proliferation of epithelial cells at the inflammatory site, which stimulate tumor initiation and growth in CRC. Furthermore, the formation of new blood vessels is essential for tumor growth and progression. These proinflammatory pathways induce the production of growth factors such as VEGF and chemokines such as IL-8 to promote angiogenesis. Additionally, upregulation of proteases facilitates tumor invasion. A crosstalk between these pathways has also been suggested. For example, regulation of IL-6 and COX-2 expression by the NF-*κ*B pathway, the proinflammatory effects of PGE_2_ through the IL-23/Th17 axis, and cytokine secretion by Th17 cells can regulate production of IL-6 and activation of STAT3. Several tumor-promoting mediators involved in these pathways function via a self-amplifying loop. For example, activation of NF-*κ*B induces the production of TNF-*α*, which can in turn function as a primary stimulus for the NF-*κ*B pathway. PPAR*δ*, downstream of the COX-2/PGE_2_ pathway, can further induce COX-2 expression and PGE_2_ production. In conclusion, these signaling pathways are widely activated in IBD and collaboratively contribute to colonic tumorigenesis by stimulating cell survival, angiogenesis, and cell invasion. Several lines of evidence have suggested that inhibition of components in these pathways is effective to suppress tumor development in colitis-associated CRC models. Better understanding of the underlying mechanisms may lead to novel targets of prevention and therapy for colitis-associated CRC.
